# Pivot burrowing of scarab beetle (*Trypoxylus dichotomus*) larva

**DOI:** 10.1038/s41598-021-93915-0

**Published:** 2021-07-16

**Authors:** Haruhiko Adachi, Makoto Ozawa, Satoshi Yagi, Makoto Seita, Shigeru Kondo

**Affiliations:** 1 Graduate School of Frontier Bioscience, Osaka University, Suita, Osaka 565-0871 Japan; 2 Graduate School of Engineering Science, Osaka University, Toyonaka, Osaka 560-8531 Japan

**Keywords:** Behavioural ecology, Animal behaviour, Entomology

## Abstract

Many organisms live in the soil but only a little is known about their ecology especially movement style. Scarab beetle larvae do not have appendages to shovel soil and their trunk is thick compared to their body length. Hence, their movement through the soil is perplexing. Here, we established the observation and analysis system of larval movement and found that the last larval instars of *Trypoxylus dichotomus* burrow in two different ways, depending on the hardness of the soil. If the soil is soft, the larvae keep their body in a straight line and use longitudinal expansion and contraction; if the soil is hard, they flex and rotate their body. It is thought that the larvae adapt to diverse soil conditions using two different excavation methods. These results are important for understanding the soil ecology and pose a challenge to engineer of newer excavation technology.

## Introduction

The soil is home to many organisms, and how they move through soil has been studied in several species (e.g., rats, lizards, snakes, nematodes, eels, spiders, ants, earthworms) by analyzing the shape of their burrows or/and using X-ray scanning technology^1–11^. Among them, earthworms have attracted much attention as a research model because they can burrow through soil without appendages, and their dynamics have recently been elucidated via an observation system using transparent superabsorbent polymers^12^. Furthermore, applications of these excavation techniques are being developed^13–15^. Earthworm morphology seems to be suitable for burrowing in terms of the drag force from the soil, as their trunk is narrow compared to their body length. However, the shape of some organisms in the soil is not suitable for burrowing. For example, the larva of the scarab beetle (*Trypoxylus dichotomus*), which spend their life in the soil until adult emergence. Their trunk is very thick compared to their body length; the transverse sectional diameter of an earthworm is approximately 2 mm^16^, whereas that of the last instar larvae is ~ 20 mm. In other words, the transverse sectional area of beetle larvae contributing to resistance from the soil is almost 100 times larger than that of a earthworm. Therefore, the burrowing mechanism must be different from that of earthworms under similar conditions. Although the beetle ecology in the soil has been reported in several studies^17,18^, little is known about how the beetle larvae move.

## Result and discussion

**Figure 1:**
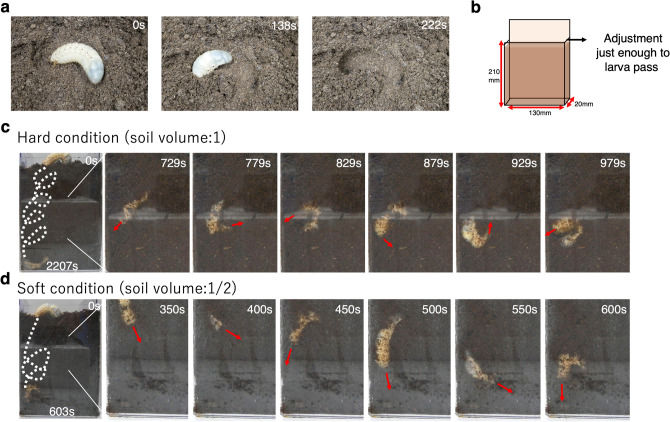
Burrowing dynamics of scarab beetle (*Trypoxylus dichotomus*) larva. (**a**) Burrowing images. After beetle is put on the soil, they can burrow in a short time (< 4 min). (**b**) A schematic of the pseudo- two dimensional (2D) observation tank. The depth was adjusted just enough for larva to pass. (**c**) Pseudo 2D observation image, before and after burrowing, and during burrowing. (**d**) The dynamics of burrowing in soft sediment [half volume soil compared to (**c**)].

Here, we analyzed the burrowing mechanisms of beetle larvae. Beetle larvae were placed on the soil surface to make sure they could burrow into the soil (Fig. 1a). In order to observe the burrowing behavior, a two-dimensional (2D) observation tank (130 × 210 ×  ~ 20 mm) was constructed (Fig. 1b); we succeeded in observing the dynamics of the larvae under a 2D soil condition (Fig. 1c, Supplementary Movie 1). The larvae burrowed by rotating themselves (Fig. 1d, Supplementary Movie 1). Rotation was observed regardless of sex. All observed individuals proceeded towards the bottom and stopped when rotating at the bottom layer (Fig. 1c).

**Figure 2:**
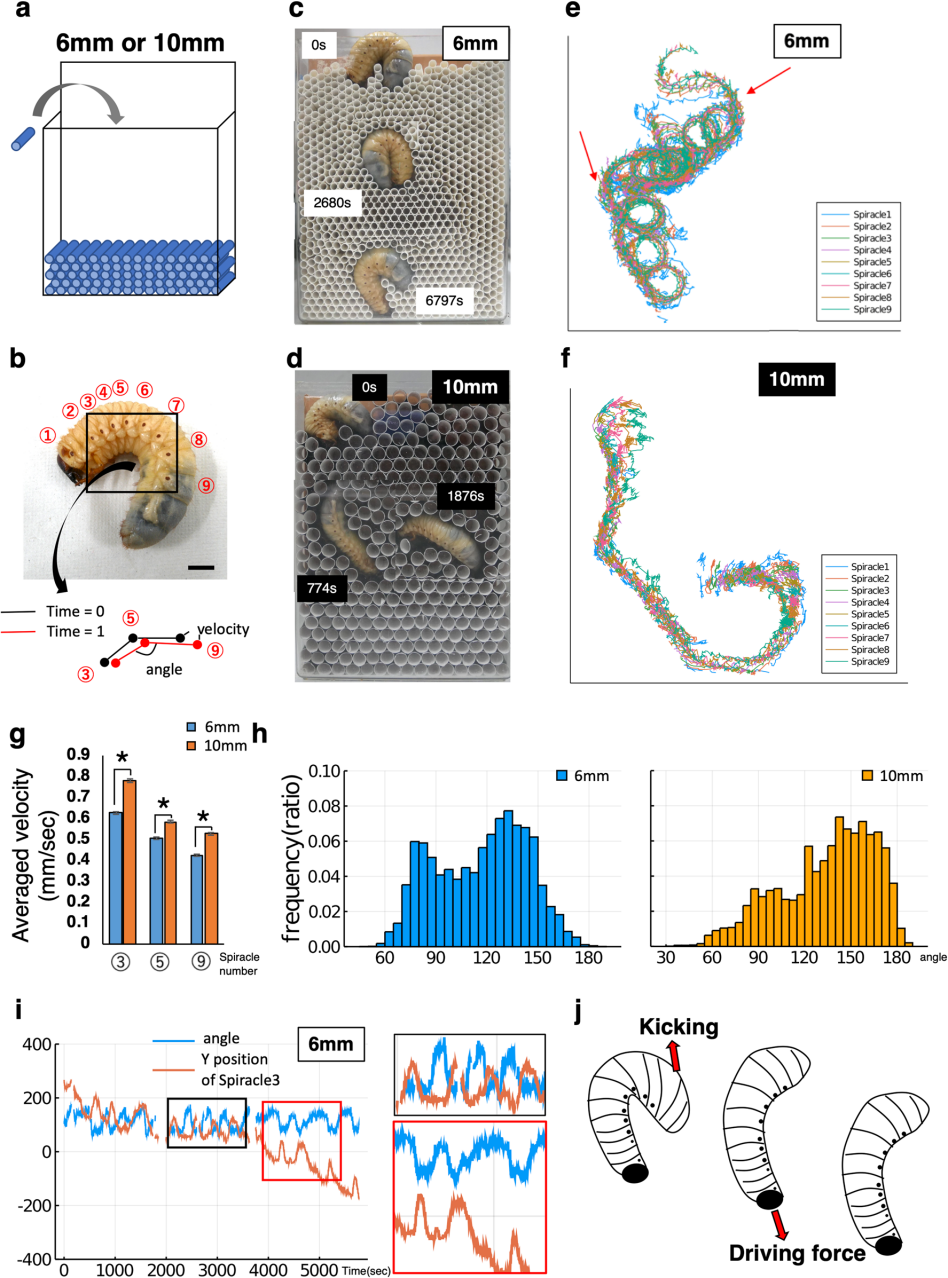
Observation system to analyze burrowing movement of scarab beetle (*Trypoxylus dichotomus*) larvae. (**a**) A schematic of the pseudo two-dimensional (2D) observation system for analysis. Cut paper straws were stacked up. (**b**) Tracking analysis scheme. Nine spiracles were tracked by the ImageJ plugin “manual tracking” (Version 1.0) from three animals in each experiment. The angle was calculated from three spiracles (3, 5, 9). (**c**, **d**) Pseudo 2D observation image burrowing in 6 mm and 10 mm cylinders. (**e**, **f**) The trajectory of nine spiracles burrowing in 6 mm and 10 mm cylinders. Red arrows show the twisting and turning points. (**g**) The average velocity of the spiracle in 6 mm and 10 mm cylinders. Each averaged over 6000 distinct velocity values from three different animals. The velocity in the 10 mm cylinder was about 1.2 times that in the 6 mm cylinder at all positions of the spiracle 3, 5, and 9. (asterisk means p < 0.05) (**h**) The histogram of the angle calculated from three spiracles (3, 5, 9) in 6 mm and 10 mm cylinders from three beetle movement. (**i**) Relationship between rotation and the velocity of spiracle 3 in 6 mm cylinders. Blue line shows the angle calculated from three spiracles (3, 5, 9), and the orange line shows the vertical position of spiracle 3 each time. (Other data in the 6 mm cylinders and 10 mm cylinders are in Fig. S2).

Next, we investigated whether the hardness of the medium being excavated could change the dynamics of burrowing. Different amounts of soil were used to compare compacted soil (relative volume of soil: 1) and non-compacted soil (relative volume of soil: 1/2). The time to reach the bottom was faster (Fig. 1d, Fig. S1A, Supplementary Movie 1) and the number of rotation was smaller when burrowing in non-compacted soft soil (Fig. 1d, Fig. S1B, Supplementary Movie 1). However, in these observation systems, there were several areas that could not be observed clearly to analyze the dynamics because the soil covered their body. Therefore, we searched for a burrowing medium that would aid in observing the burrowing dynamics clearly. We constructed a complete 2D observation system by stacking cylindrical paper straws of two diameters cut into 2 cm segments and observed in detail the movements and changes in shape of the larvae there (Fig. 2a, b). By changing the diameter of the straw (using 6 mm and 10 mm diameter straws), the area of the lumen changes, which is thought to have the similar effect as soil compaction changes.

As a result, we could observe quite clearly how the burrowing larvae rotate. Through the 6 mm straws, the larvae proceeded towards the bottom by rotating and stopped at the bottom layer just as in the hard soil (Fig. 2c, Supplementary Movie 3). In 10 mm straws condition, the larvae did not move in a straight line to the bottom layer, and when they did reach the bottom, they did not stay there, but moved to the upper layer, contrary to their behaviour in soft soil (Fig. 2d, Supplementary Movie 5). Although beetle larvae are known to be able to change their burrowing direction depending on CO_2_ concentration^17^, their movement may also be motivated by the size of the surrounding particles or by sensing other factors that vary with size.

We analyzed the movie of larval dynamics using Fiji’s Plugin “manual tracking” (version 1.0) to obtain a set of coordinates for the larval spiracles. Beetle larvae have nine spiracles, which were used as indicators in the tracking analysis (Figs. 2b,e,f, S1, Supplementary Movie 4 and 6) (some parts, where the spiracles were not visible, were skipped.). Larvae could twist and turn their bodies in the 2D environment (Fig. 2e, red arrow). We first investigated the velocity of the spiracles. Movement of anterior parts was faster than posterior parts, for example, spiracle 3 was approximately 1.2 times faster than spiracle 5, and spiracle 5 was also approximately 1.2 times faster than spiracle 9. And the velocity in the 10 mm cylinder was approximately 1.2 times higher than that in the 6 mm cylinder at all positions of spiracles 3, 5, and 9. The differences were statistically significant. (Fig. 2g). This velocity tendency (6 mm vs 10 mm) corresponds to the tendency in the soil compaction experiment (with vs without compaction). In addition, the change in angle between the three spiracles (3, 5, 9) was measured (Fig. 2h). Although there were two peak angles under both straw medium conditions, the position of the peaks was different. In 6 mm straws, the first peak was ~ 80° and the second peak was ~ 130°. In 10 mm straws, the peaks were at ~ 90° and ~ 150°. The frequency of the peaks was also different. In the 6 mm straws, the frequency in the range of 60°–100° was 31.1%. In the 10 mm straws, the frequency in the range of 60°–100° was 17.0%. Thus, the first peak distribution was reduced by approximately 46% (Fig. 2h). The two peaks indicate two modes of larval shape; the first peak indicates C-shaped shape, and the second peak indicates straight shape. The two peaks show indicate two modes of larval morphology shape; the first peak indicates C-shaped shape, and the second peak indicates straight shape. It is assumed that there exists a relationship between larval shape and the presence or absence of pivot movement.

In both soil and straw experiments, the larvae were less likely to rotate under conditions of faster burrowing and more likely to rotate under conditions of slower burrowing. Apparently, there is a relationship between burrowing speed and the presence or absence of pivot movement. It seems that larval pivot burrowing can change with the surrounding environment.

Next, we examined the relationship between rotation position and larval shape. To investigate the location of rotation, we plotted the time transition of the Y-position of spiracle 3. With rotation, the Y-position fluctuated up and down repeatedly. Time series data of the angle were plotted. Angle and Y-position were inversely related (Figs. 2i, S2, S4). This suggests that rotational burrowing may contribute to the larvae pushing (kicking) the soil with their tails to move downward (Fig. 2j). Another possible reason is that the rotation and pivoting motion causes local fluidization of the soil, thereby reducing the resistance^19^. The reason for the lack of a clear inverse relationship before reaching a certain depth is thought to be that the straw was not heavy enough to provide a drag force when pushed with the buttocks. Although the tiger beetle larvae also burrow in the soil^20,21^, the shape of the hole is straight, and it is assumed that they do not burrow with rotation like the scarab beetle larvae. The tiger beetle larvae are not C-shaped like the scarab beetle larvae^20,21^. There is a possibility that larval morphology may cause rotation. In the future, comparisons with the tiger beetle can facilitate ecological evolutionary developmental biology (Eco-Evo-Devo) research.

In this study, we established a 2D observation system for beetle larvae and analyzed how they move. As a result, we discovered that scarab beetle larvae burrow by rotating. Burrowing dynamics changed when the hardness and size of the soil particles were changed. In addition, the relationship between larval shape and timing of rotation was clarified, suggesting that rotated movement may be conducive to displacing the soil to move downwards. A 2D observation system was used in this study; it is unclear whether the same behavior would be observed in a 3D environment. X-ray CT technology enables observations in a 3D environment, but the slow scanning speed of current technology makes it difficult to track behaviour in real time. In the future, with faster scanning, we will be able to observe larval behaviour in a 3D environment. The rotational movement may be an effective way of burrowing by repeating simple movements. The significance of this rotational motion needs to be confirmed by reproducing it in a robot. Some studies have investigated the development of excavation robots that apply the burrowing mechanisms of animals (e.g., inchworms, shellfish, and earthworms)^15,22–24^. The application of the burrowing mechanism of beetle larvae may also contribute to the development of new excavation technologies.

The rotational movement is caused by the shape of the larvae. Comparison of the larvae with those of other soil insects, such as the tiger beetle larvae^20,21^, which burrows linearly, would facilitate Eco-Evo-Devo research. Additionally, the results of studies on the frequency, position, and direction of the rotation can be applied in the field of neuroethology.

## Materials and methods

### Insects

The last instars of Japanese rhinoceros beetle (*Trypoxylus dichotomus*) were commercially purchased (Finebeetle, Ehime, Japan) and kept individually in 500–1000 mL bottles filled with rotted wood flakes at 10–15 °C until use.

### Observation of burrowing

The larvae were moved to a room with a temperature of 23 °C and could acclimatize for at least 30 min before the burrowing experiment was conducted. Larval movements in the glass tank (6 × 21 × 2 cm) were captured by a time-lapse camera (DMCGX7MK2WK, Panasonic, Osaka, Japan). The larvae were incubated in a humus mat (Kanzen, Tsukinoyakinokoen, Gunma, Japan) and paper straws (6 mm, Shimojima, Tokyo, Japan) (10 mm, Zone Plus, Shiga, Japan) were cut into ~ 2 cm pieces for visualization. Each experiment was conducted three times.

### Analysis of tracking

From the data obtained by time-lapse photography, the coordinates of the spiracles present in each body segment of the larvae were obtained for each time series using Fiji’s macro manual tracking (version1.0). The data were skipped when the spiracles were unclear. Based on the size of the tank, the distance per pixel was calculated and used for subsequent determination of velocity between the spiracles. In the time lapse, the images were taken every second, so the displacement of each image of each trachea was directly used as the velocity. The angle was calculated from the positional information of the three spiracles. All analysis after obtaining the positional information was done in Julia language (version 1.5).

### Statistical analysis

In single comparisons, we applied Student's t test, and we considered p-value of less than 0.05 to be a statistical significance.

The data for which correlation coefficients were calculated were subjected to test for no correlation. and we considered p-value of less than 0.05 to be a significant correlation.

## Supplementary Information

Supplementary Information 1.

Supplementary Dataset.

Supplementary Movie 1.

Supplementary Movie 2.

Supplementary Movie 3.

Supplementary Movie 4.

Supplementary Movie 5.

Supplementary Movie 6.

## References

[1] Maladen RD, Ding Y, Li C, Goldman DI (2009). Undulatory swimming in sand: Subsurface locomotion of the sandfish lizard. Science.

[2] Maladen RD, Ding Y, Umbanhowar PB, Kamor A, Goldman DI (2011). Mechanical models of sandfish locomotion reveal principles of high performance subsurface sand-swimming. J. R. Soc. Interface.

[3] Sharpe SS, Koehler SA, Kuckuk RM, Serrano M, Vela PA, Mendelson J, Goldman DI (2015). Locomotor benefits of being a slender and slick sand swimmer. J. Exp. Biol..

[4] Jung S (2010). Caenorhabditis elegans swimming in a saturated particulate system. Phys. Fluids.

[5] Herrel A (2011). Burrowing and subsurface locomotion in anguilliform fish: behavioral specializations and mechanical constraints. J. Exp. Biol..

[6] Hils JM, Hembree DI (2015). Neoichnology of the burrowing spiders *Gorgyrella inermis* (Mygalomorphae: Idiopidae) and Hogna lenta (Araneomorphae: Lycosidae). Palaeontol. Electron..

[7] Monaenkova D (2015). Behavioral and mechanical determinants of collective subsurface nest excavation. J. Exp. Biol..

[8] Joschko M (1991). A non-destructive method for the morphological assessment of earthworm burrow systems in three dimensions by X-ray computed tomography. Biol. Fertil. Soils.

[9] Jégou D, Cluzeau D, Wolf HJ, Gandon Y, Tréhen P (1997). Assessment of the burrow system of *Lumbricus terrestris*, *Aporrectodea giardi*, and *Aporrectodea caliginosa* using X-ray computed tomography. Biol. Fertil. Soils.

[10] Jégou D, Hallaire V, Cluzeau D, Tréhen P (1999). Characterization of the burrow system of the earthworms *Lumbricus terrestris* and *Aporrectodea giardi* using X-ray computed tomography and image analysis. Biol. Fertil. Soils.

[11] Thomas HG (2009). Burrow architecture and digging activity in the Cape dune mole rat. J. Zool..

[12] Kudrolli A, Ramirez B (2019). Burrowing dynamics of aquatic worms in soft sediments. Proc. Natl. Acad. Sci. USA.

[13] Kubota T, Nagaoka K, Tanaka S, Nakamura T (2007). Earth-worm typed drilling robot for subsurface planetary exploration. IEEE Int. Conf. Robot. Biomimet..

[14] Tadami, N., Nagai, M., Nakatake, T., Fujiwara, A., Yamada, Y., Nakamura, T., Yoshida, H., Sawada, H. & Kubota, T. Curved Excavation by a Sub-seafloor Excavation Robot. *IEEE/RSJ International Conference on Intelligent Robots and Systems (IROS)/Workshop on Machine Learning Methods for High-Level Cognitive Capabilities in Robotics.* 4950–4955 (2017).

[15] Isaka K (2019). Development of Underwater Drilling Robot Based On Earthworm Locomotion. IEEE Access.

[16] Quillin KJ (1999). Kinematic scaling of locomotion by hydrostatic animals: Ontogeny of peristaltic crawling by the earthworm *Lumbricus terrestris*. J. Exp. Biol..

[17] Kojima W (2015). Attraction to carbon dioxide from feeding resources and conspecific neighbours in larvae of the rhinoceros beetle *Trypoxylus dichotomus*. PLoS ONE.

[18] Kojima W, Takanashi T, Ishikawa Y (2012). Vibratory communication in the soil: Pupal signals deter larval intrusion in a group-living beetle *Trypoxylus dichotoma*. Behav. Ecol. Sociobiol..

[19] Winter AG, Deits RLH, Hosoi AE (2012). Localized fluidization burrowing mechanics of *Ensis directus*. J. Exp. Biol..

[20] Gilbert C (1989). Visual determinants of escape in tiger beetle larvae (Cicindelidae). J. Insect Behav..

[21] Shelford VE (1908). Life-histories and larval habits of the tiger beetles (Cicindelidæ). J. Linn. Soc. Lond. Zool..

[22] Zhang, W. W., Jiang, S. Y., Shen, Y., Li, P. & Chen, H. Z. Development of an inchworm-type drilling test-bed for planetary subsurface exploration and preliminary experiments. *2016 IEEE International Conference on Robotics and Biomimetics (Robio)*, 2187–2191 (2016).

[23] Winter, V. A. G., Deits, R. L. H., Dorsch, D. S., Hosoi, A. E. & Slocum, A. H. Teaching RoboClam to Dig: The Design, Testing, and Genetic Algorithm Optimization of a Biomimetic Robot *IEEE/RSJ International Conference on Intelligent Robots and Systems.* (2010).

[24] Koller-Hodac, A., Germann, D. P., Gilgen, A., Dietrich, K., Hadorn, M., Schatz, W. & Hotz, P. E. Actuated Bivalve Robot Study of the Burrowing Locomotion in Sediment *IEEE International Conference on Robotics and Automation (ICRA).* 1209–1214 (2010).

