# Supplementary material for: Pivot burrowing of scarab beetle (Trypoxylus dichotomus) larva
Source: Sci Rep. 2021 Jul 16;11:14594. doi: 10.1038/s41598-021-93915-0 (PMC8285476; doi:10.1038/s41598-021-93915-0)
Supplement: Supplementary file 1 — Supplementary Information 1. [file 41598_2021_93915_MOESM1_ESM.pdf]

Pivot burrowing of scarab beetle (*Trypoxylus dichotomus*) larva

Haruhiko Adachi[1]*, Makoto Ozawa[2], Satoshi Yagi[2], Makoto Seita[1], Shigeru Kondo[1]

[1.] Graduate School of Frontier Bioscience, Osaka University, Suita, Osaka, 565-0871, Japan

[2.] Graduate School of Engineering Science, Osaka University, Toyonaka, Osaka 560-8531, Japan

[*] Corresponding author and address. E-mail: hrhk.adachi@gmail.com

Figure S1. Difference in the burrowing speed and the degree of rotation between soft and hard conditions

Figure S2. Other larval dynamics in two-dimensional condition 6 mm and 10 mm cylinder

Figure S3. Relationship between rotational movement and larval morphology in the other larva

Figure S4. Relationship of the slope between rotation movement and larval morphology

Figure S5. Relationship between time difference (for determining slope) and correlation coefficient

Supplementary Movie 1 Burrowing dynamics of *Trypoxylus dichotomus* beetle larva in two dimensional hard soil

Supplementary Movie 2 Burrowing dynamics of *Trypoxylus dichotomus* beetle larva in two dimensional soft soil

Supplementary Movie 3 Burrowing dynamics of *Trypoxylus dichotomus* beetle larva in a two dimensional 6 mm cylinder

Supplementary Movie 4 Tracking of spiracles of burrowing *Trypoxylus dichotomus* beetle larva in a two dimensional 6 mm cylinder

Supplementary Movie 5 Burrowing dynamics of *Trypoxylus dichotomus* beetle larva in a two dimensional 10 mm cylinder

Supplementary Movie 6 Tracking of spiracles of burrowing *Trypoxylus dichotomus* beetle larva in a two dimensional 10 mm cylinder

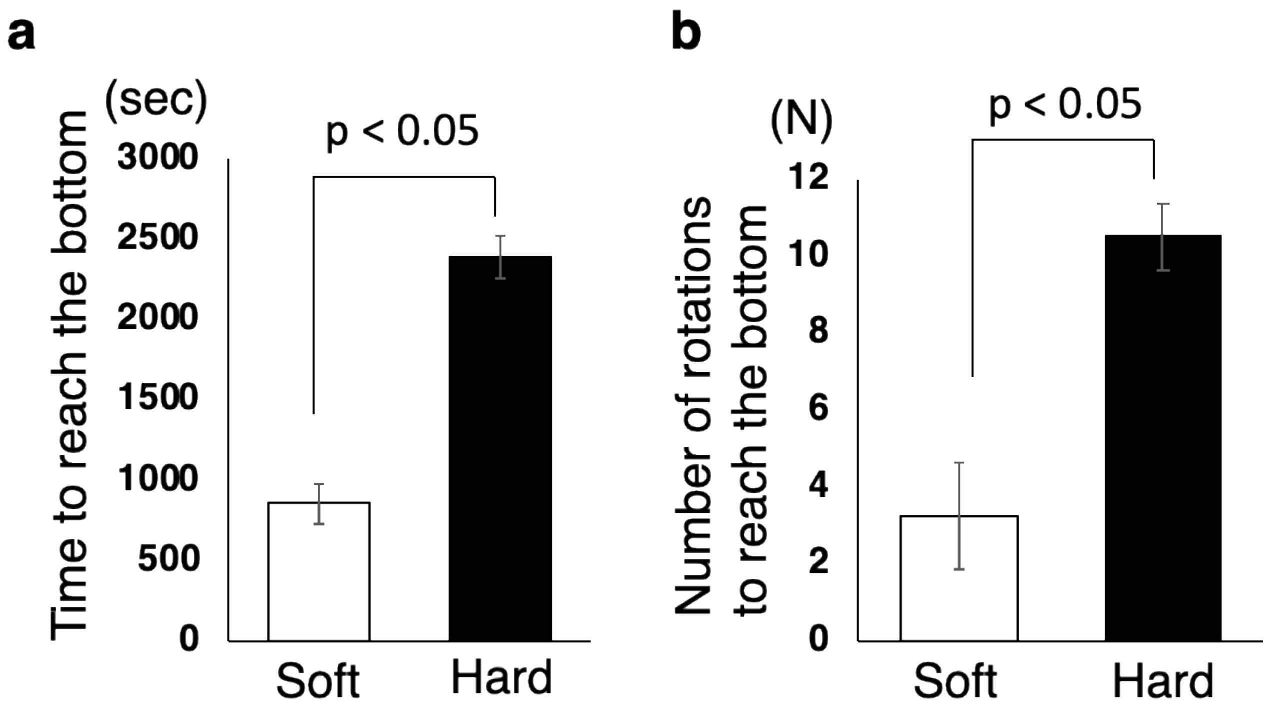

Figure S1. Difference in the burrowing speed and the degree of rotation between soft and hard conditions

(a) The time to reach the bottom was analyzed from four samples of each treatment group. The time was significantly faster in the soft condition ($p<0.05$). (b) The number of rotations to reach the bottom was analyzed from four samples of each treatment group. The number was significantly higher in the hard condition ($p<0.05$).

6 mm cylinder other sample

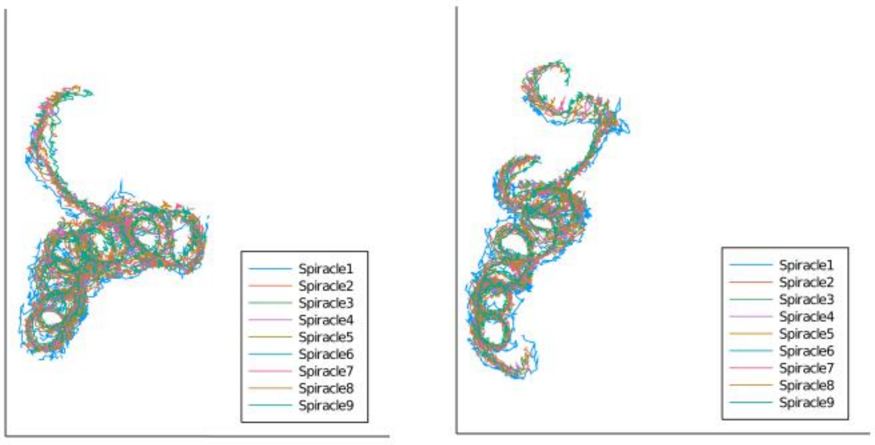

10 mm cylinder other sample

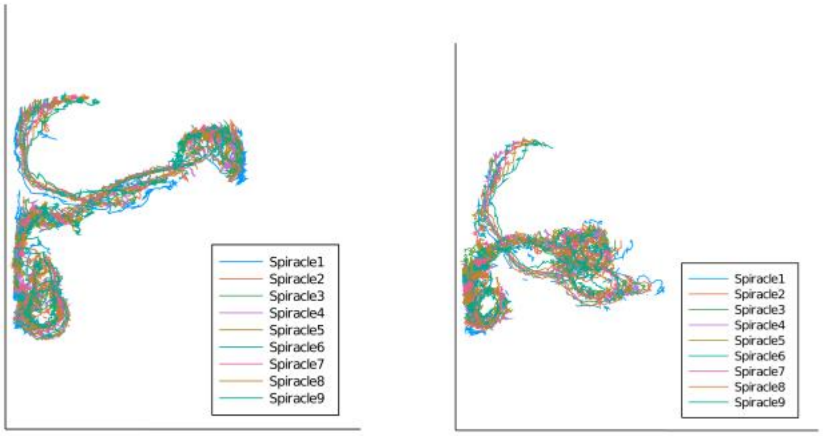

Figure S2. Other larval dynamics in 2D 6mm and 10mm cylinder

Other sample trajectory of nine spiracles burrowing in 6mm and 10mm cylinder conditions.

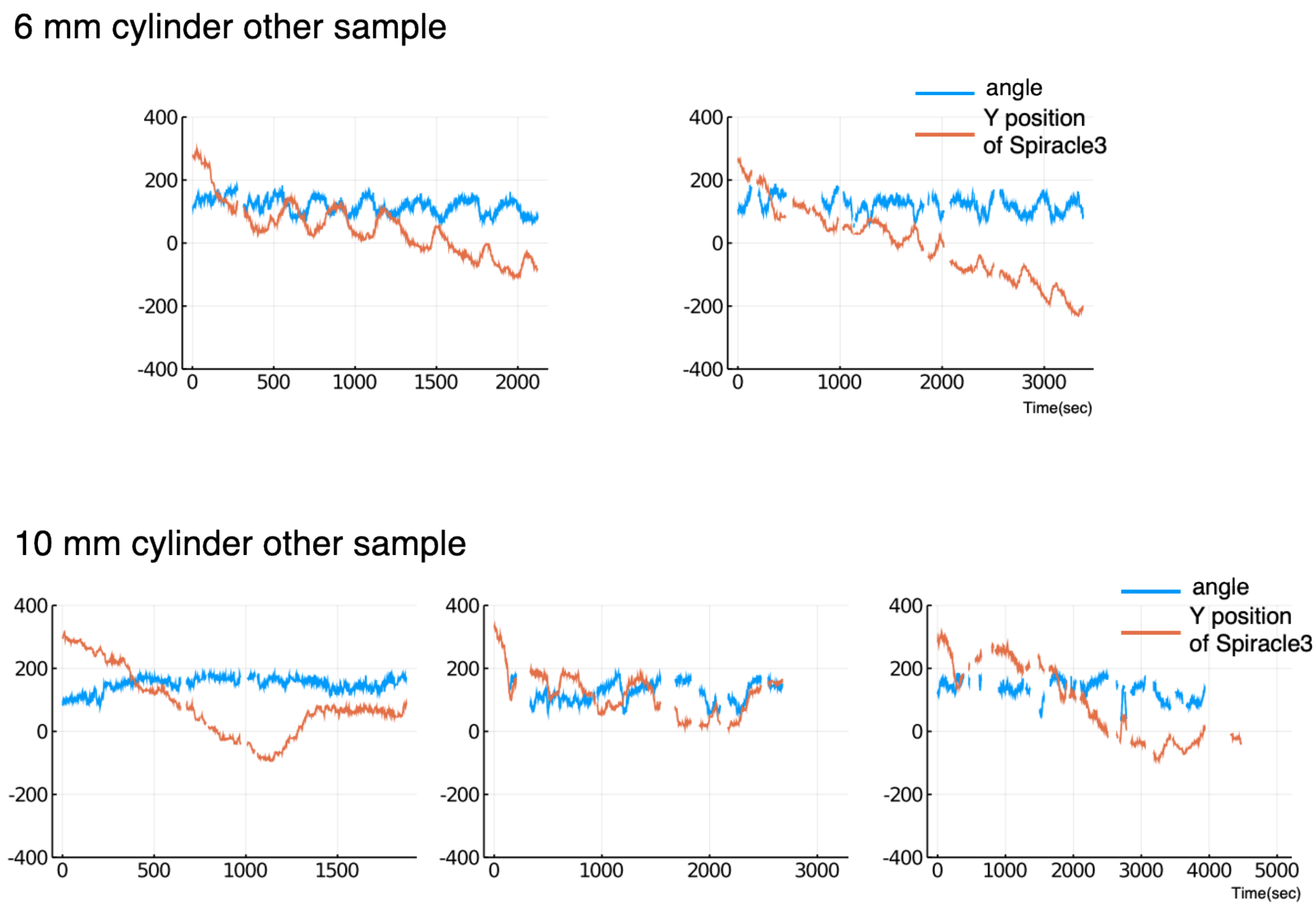

Figure S3. Relationship between rotation movement and larval morphology in the other larva

Other samples relationship between the rotation and the velocity of spiracle 3 in 6 and 10 mm cylinder conditions. Blue line shows the angle calculated from three spiracles (3,5,9) and orange line shows the vertical position of spiracle 3 at each time.

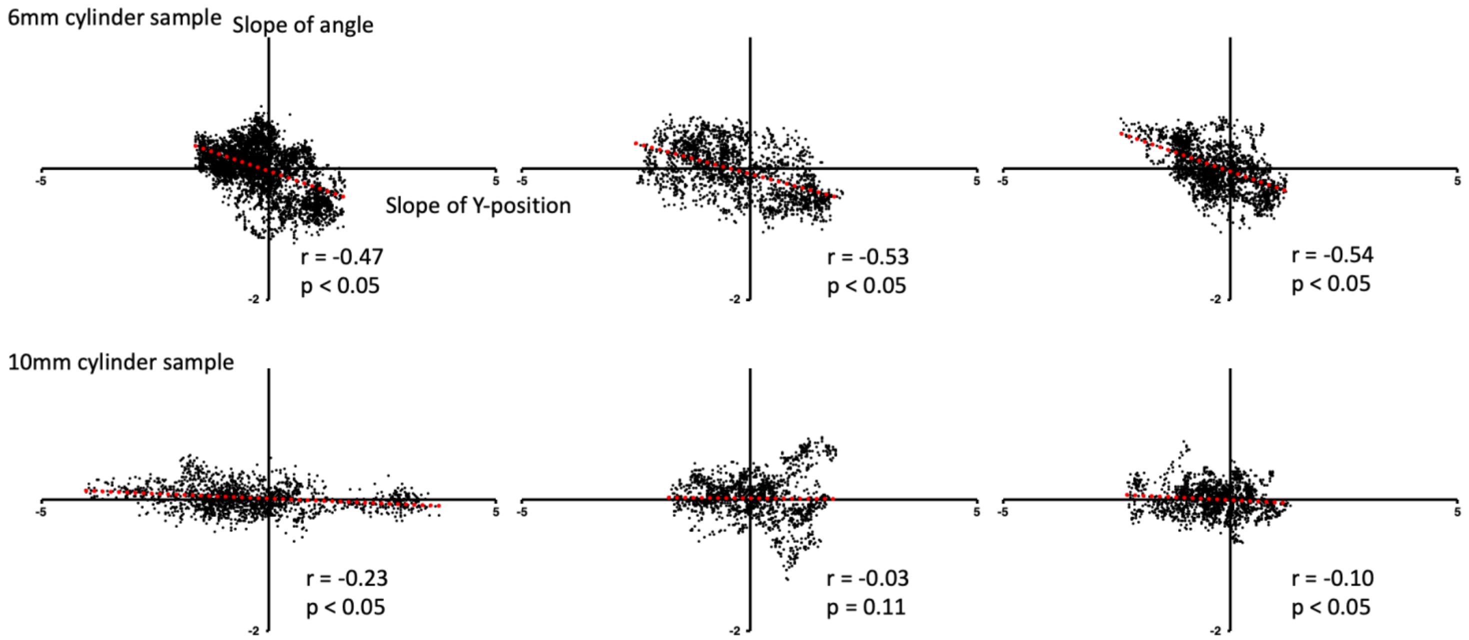

Figure S4. Relationship of the slope between rotation movement and larval morphology in the other larva

We calculated the respective slopes of the time series changes in Angle $\theta(t)$ and Y-position $y(t)$ in Fig. 2i and Fig. S3 ($slope = \frac{\theta(t+\Delta t) - \theta(t)}{\Delta t}$ or $\frac{y(t+\Delta t) - y(t)}{\Delta t}$ ($\Delta t = 100$ s)). They showed inverse relationship and it was statistically significant in 6 mm cylinder sample (p<0.05). In contrast, no clear relationship was found in the 10 mm cylinder sample.

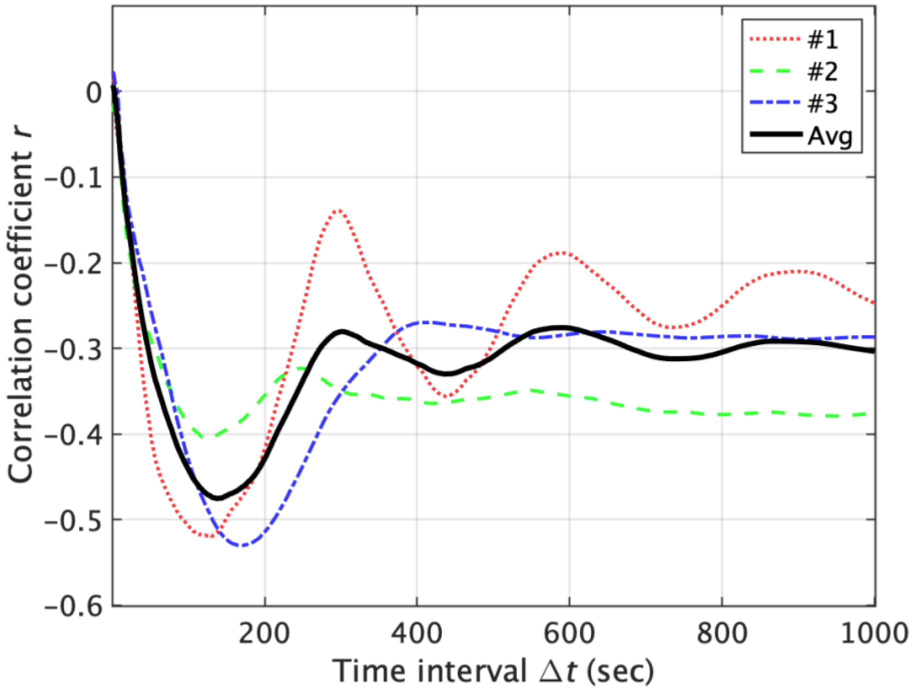

Figure S5. Relationship between time interval (for determining slope) and correlation coefficient *r*.

We calculated how the coefficient r changes with time interval Δt (for determining slope) from linearly-interpolated data. When the time interval was small, the correlation coefficient r was close to zero. This is thought to be due to noise in the data. We chose a time interval of 100 seconds, which is as close to the original data as possible and also reduces the effect of noise, to determine the slope, for Fig.S4.
